# Crosstalk between enteric serotonergic neurons and colorectal cancer stem cells to initiate colorectal tumorigenesis

**DOI:** 10.3389/fonc.2022.1054590

**Published:** 2022-11-02

**Authors:** Jiamei Jia, Mengmeng Wang, Shuqiao Xing, Zhihui Huang, Yuanyuan Jiang

**Affiliations:** ^1^ School of Pharmacy, Hangzhou Normal University, Hangzhou, China; ^2^ Key Laboratory of Elemene Class Anti-Cancer Chinese Medicines; Engineering Laboratory of Development and Application of Traditional Chinese Medicines; Collaborative Innovation Center of Traditional Chinese Medicines of Zhejiang Province, Hangzhou Normal University, Hangzhou, China

**Keywords:** colorectal cancer, colorectal cancer stem cells, 5-hydroxytryptamine, Wnt/β-catenin, isovalerate

## Introduction

Colorectal cancer (CRC) is the second most common cause of cancer-related deaths and the third most common cancer worldwide (1). Although treatments such as endoscopic and local surgical resection, local ablative treatment of metastases, and palliative chemotherapy have prolonged the overall survival of patients with advanced disease, CRC still exhibits a poor prognosis and a low long-term survival rate (2). Colorectal cancer stem cells (CSCs) are a small subpopulation of cells with self-renewal and differentiation ability in CRC (3). Aberrant hyperactivation of some signaling pathways in CSCs such as Wnt/β-catenin, Notch, and Hedgehog may induce uncontrolled cell proliferation and abnormal differentiation, leading to tumorigenesis in a tissue-specific manner (4). However, the molecular mechanism of colorectal CSCs regulation remains to be explored.

5-hydroxytryptamine (5-HT), also known as serotonin, is mostly produced in the intestines (5). It is reported that the biosynthesis and secretion of 5-HT were found to be upregulated in colorectal tumor tissues, and overproduced 5-HT enhanced NLRP3 inflammasome activation in macrophages, which may further promote 5-HT biosynthesis (6). Moreover, the gut microbiota is a complex community of microorganisms that live in the digestive tracts of humans and animals, which have many functions in the human body, such as enhancing the immune system, participating in digestion and metabolism, and influencing brain–gut communication (7–9). Ecological dysbiosis of the intestinal flora may be closely associated with the progression of gastrointestinal diseases such as inflammatory bowel disease and CRC (10, 11). Can 5-HT regulate colorectal CSCs and participate in the progression of CRC? Is the gut microbiota also involved in this process? Recently, Zhu et al. published a research article in *Neuron* entitled “5-Hydroxytryptamine produced by enteric serotonergic neurons initiates colorectal cancer stem cell self-renewal and tumorigenesis” (12) and investigated these issues.

## Colorectal cancer-associated microbiota metabolite isovalerate initiates 5-HT production in serotonergic neurons to drive colorectal cancer stem cell self-renewal and colorectal tumorigenesis

To investigate whether 5-HT has an effect on colorectal CSC physiology, 11 neurotransmitters were screened by a sphere formation assay to detect its self-renewal *in vitro*. Authors found that 5-HT remarkably promoted the sphere formation of GFP^high^ cells, which were the colorectal CSCs from *Lgr5* ^GFP^ mice with CRC induced by dextran sodium sulfate/azoxymethane (DSS/AOM). Similar effects were found in human LGR5^GFP+^ CSCs as well. In *in vivo* assays, authors used tryptophan hydroxylase (Tph) inhibitor parachlorophenylalanine (pCPA) to inhibit 5-HT production and serotonin transporter (*Sert*) KO mice to consistently elevate 5-HT production to demonstrate the role of 5-HT in colorectal CSC self-renewal and tumorigenesis. 5-HT was reported to activate acetylcholine (Ach) in the intestines and promote the proliferation of crypt cells (13). However, researchers tested and proved that 5-HT promoted self-renewal of colorectal CSC and colorectal tumorigenesis independent of Ach both *in vitro* and *in vivo*.

In the gut, both enterochromaffin (EC) cells in the villus, which contain Tph1, and serotonergic neurons in the myenteric plexus, which express Tph2, are responsible for the biosynthesis of 5-HT. Researchers then tried to explore the source of 5-HT for colorectal CSC self-renewal and tumorigenesis. Results showed that *TPH1* mRNA was lower while *TPH2* mRNA was increased in colorectal tumors than in non-tumor tissue. Its TPH2 expression but not TPH1 was positively correlated with intratumoral 5-HT levels. Furthermore, TPH2^+^ cells were enriched close to the tumors in human colorectal tumors, and Tph2^+^ cells were enriched in the “tumor root region” where the tumor originated in mouse CRC tissues. Moreover, authors demonstrated that *Tph2* KO mice displayed reduced colorectal tumorigenesis, reduced number of CSCs, weaker CSC proliferation capacity, and decreased 5-HT level upon DSS/AOM treatment, while 5-HT could rescue the defect of CSCs self-renewal with supernatants from *Tph2* KO neurons as expected. What is more, Tph2^+^ serotonergic neurons specialized and deleted by diphtheria toxin (DT) administration in *Tph2* ^DTR^ mice further demonstrated that CSC self-renewal and colorectal tumorigenesis needed 5-HT from Tph2^+^ serotonergic neurons.

To explore whether 5-HT receptors on the colorectal CSCs were engaged in 5-HT signaling for CSC self-renewal and colorectal tumorigenesis, 5-HT receptors were silenced in human colorectal CSCs, while only *HTR1B*, *HTR1D*, or *HTR1F* knockdown could significantly reduce the self-renewal of CSCs. By establishing *HTR1B*, *HTR1D*, and *HTR1F* triple knockout (TKO) cells and *Htr1b*, *Htr1d*, *and Htr1f* TKO mice, authors demonstrated that 5-HT signaling initiated colorectal CSC self-renewal and tumorigenesis by engaging 5-HT receptors HTR1B, HTR1D, and HTR1F both *in vivo* and *in vitro*. To investigate the molecular mechanism of 5-HT-promoted colorectal CSC self-renewal, authors first tested and excluded the G protein subunit alpha i1 (HTR1-GNAI) pathway, a typical pathway in which 5-HT exerted its role (14), while the Wnt/β-catenin signaling pathway was found to be significantly activated with 5-HT treatment after examining several signaling pathways. Through coimmunoprecipitation followed by mass spectrometry, authors found that AXIN1, which acts as a core component for the β-catenin degradation complex (15), was a candidate interaction protein for HTR1B. Further experimental results indicated that 5-HT enhanced the interaction of HTR1B/1D/1F with AXIN1 and then activated Wnt/β-catenin signaling *via* blocking β-catenin degradation. In addition, using specific inhibitor PNU-74654 to inactivate Wnt/β-catenin signaling could effectively eliminate 5-HT-induced sphere formation. Apc^min/+^ mice continuously activate Wnt/β-catenin signaling to initiate CRC formation (16), but 5-HT failed to promote the propagation of Apc^min/+^ CSCs. These findings uncovered that 5-HT signaling promoted self-renewal of colorectal CSCs through activating the Wnt/β-catenin pathway.

As increased Tph2 expression was found in intestinal tissue from DSS/AOM-treated mice, how was *Tph2* transcription initiated in serotonergic neurons for 5-HT production? Authors found that Tph2 expression was promoted when fecal microbiota transplantation (FMT) from DSS/AOM-induced mice to normal mice was carried out to remodel their gut microbiota, suggesting that it was CRC-associated microbiota that promoted Tph2 expression. By selecting and testing microbiota metabolites that were highly expressed in human and mouse CRC tissue, researchers found that isovalerate (IVA) could significantly promote Tph2 expression in myenteric plexus cells. The fact that IVA enhanced 5-HT was further verified. In addition, IVA was also demonstrated to augment Wnt/β-catenin target genes in colon tissue. In molecular mechanism exploration, authors discovered that the *Tph2* promoter was activated in DSS/AOM-treated myenteric plexus cells and IVA acted directly on enteric neurons to increase Tph2 expression. IVA inhibited the enrichment of nucleosome remodeling and deacetylation (NuRD) complex, which could repress the transcription of target genes, onto the *Tph2* promoter to initiate Tph2 transcription, which was further verified.

At last, the researchers provided evidence that targeting the 5-HT–Wnt axis could effectively intervene against CRC propagation and metastasis. In addition, the author showed that 5-HT levels were highly expressed in human colorectal tumors and related to the clinical prognosis of CRC patients. 5-HT^high^ primary colorectal tumors indeed displayed increased colorectal CSC ratios; enhanced sphere formation and metastasis capacity with increased LGR5 expression; enhanced AXIN1 membrane translocation, robust β-catenin expression, and nuclear translocation; and enhanced expression of Wnt/β-catenin target genes compared to 5-HT^low^ tumors.

## Discussion

**Figure 1:**
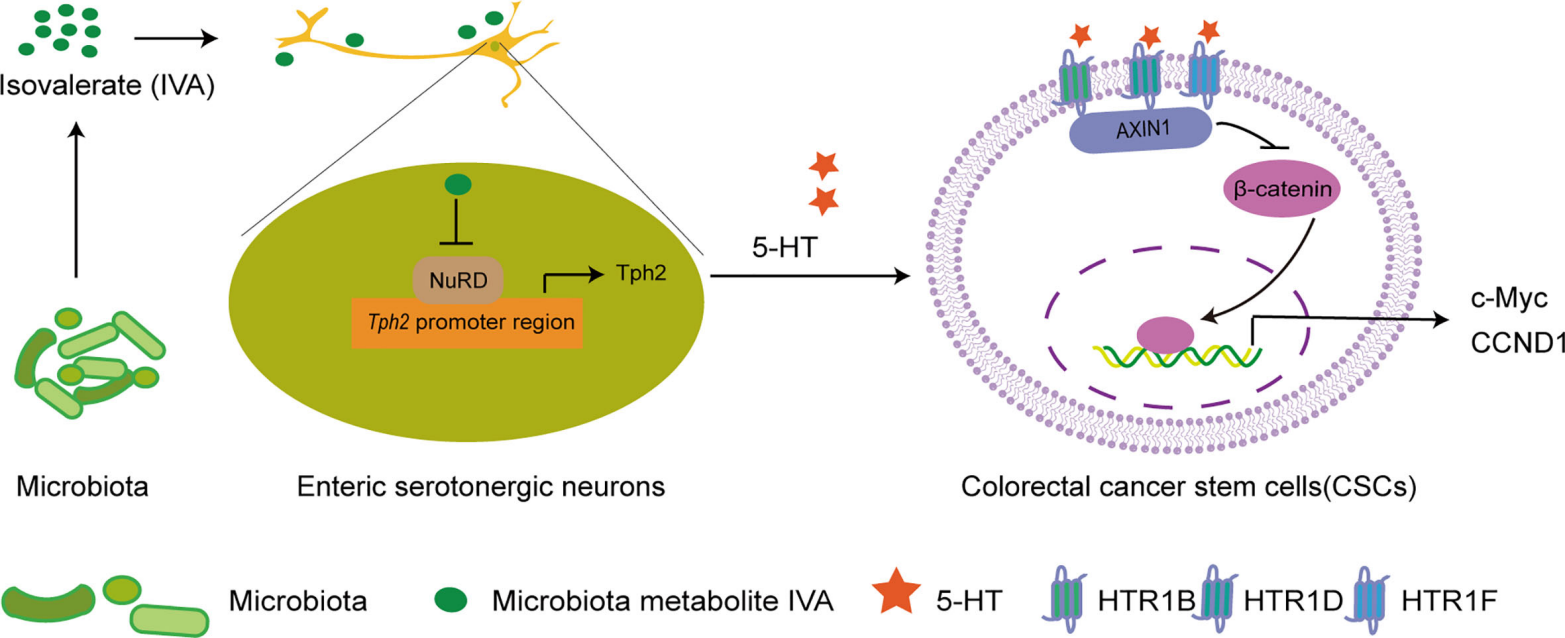
IVA stimulates 5-HT production in enteric serotonergic neurons to drive colorectal CSC self-renewal and tumorigenesis *via* 5-HT–Wnt axis. **(A)** Microbiota metabolite IVA inhibits the enrichment of NuRD complex onto *Tph2* promoter to initiate Tph2 expression, leading to the production of 5-HT in enteric serotonergic neurons. By engaging with the receptors HTR1B, HTR1D, and HTR1F on colorectal CSCs, 5-HT promotes the interaction between AXIN1 and HTR1B/1D/1F, thus promoting the stability of β-catenin, activating the Wnt/β-catenin signaling pathway, and enhancing the expression of Wnt/β-catenin signaling target genes such as c-MYC and CCND1, leading to the self-renewal of colorectal CSCs and colorectal tumorigenesis. IVA, isovalerate; CSC, cancer stem cell.

In summary, Zhu and colleagues revealed that CRC-associated microbiota metabolite IVA promoted 5-HT production from serotonergic neurons by inhibiting NuRD complex onto *Tph2* promoter to initiate Tph2 expression; by engaging 5-HT receptor HTR1B/1D/1F, 5-HT promoted colorectal CSCs self-renewal through activating Wnt/β-catenin signaling pathway and thus initiating CRC (**Figure 1**). The results are solid and compelling. More importantly, these also make us aware of the intact crosstalk among enteric microbiota, enteric nervous system, and colorectal CSCs to CRC initiation, thus pointing new perspective for CRC treatment.

Serotonergic neurons were found aggregated in proximity to tumors and affected tumor growth; however, it is still unknown whether they project axons into the tumors. This study indicated that serotonergic neurons act on the CRC stem cells, which are mucosal cells before metastasis, by releasing 5-HT. This is consistent with previous findings that serotonergic neurons of the myenteric plexus do not project to the mucosa (13, 17) but explain how they affect colorectal CSCs. In addition, another work by Zhu et al. published in *Cell Research* also elaborated on the self-renewal of intestinal stem cells *via* 5-HT from enteric serotonergic neurons in a macrophage-dependent way regulated by gut microbiota metabolite valeric acid (18).

As 5-HT–Wnt signaling is implicated in the promotion of colorectal CSCs self-renewal and CRC tumorigenesis and metastasis, blocking 5-HT-Wnt signaling may be a potential strategy for CRC therapy. Drugs that influence the effects of 5-HT may be used to fight CRC, while 5-HT enhancement-related drugs should be avoided. In addition, the Wnt/β-catenin pathway plays a key role in embryonic development and adult tissue homeostasis, and dysregulation of Wnt/β-catenin signaling generally leads to many serious diseases, both cancerous and non-cancerous; the canonical Wnt pathway has emerged as a very attractive therapeutic target in recent years (15).

Intestinal flora and its metabolite play an important role in CRC carcinogenesis. Wong et al. have reported that the fecal microbiota from patients with CRC can promote tumorigenesis in germ-free mice and mice given a carcinogen (19). Metabolite IVA is a branched-chain fatty acid produced by bacterial fermentation from leucine (20). A previous study has shown that fecal IVA concentrations in newborns are at low levels but upregulated after 6 months of age; the mean IVA levels remain at this level thereafter but with individual differences (21). However, fecal metabolomic data on samples from participants showed that IVA increased gradually from stage 0 CRC to stage III/IV CRC (22), indicating that IVA expression was positively correlated to the CRC severity. The findings in this *Neuron* work revealed the relationship between IVA and enteric serotonergic neurons in promoting 5-HT production and gave us a deeper awareness of the role of intestinal flora and its metabolite in regulating CSC stemness and CRC carcinogenesis. Interference in the production of CRC-associated microbiota metabolite IVA for clinical CRC treatment may be another strategy for CRC therapy.

However, several issues still need to be further studied. Authors displayed that the expression of TPH1 is decreased in tumors compared to normal tissues and excluded EC cells as the 5-HT source for colorectal CSCs self-renewal. However, reduced tumor number and load of DSS/AOM-induced tumors were also observed in *Tph1* KO mice (without statistical significance), and this reduction was significant in DKO (*Tph1* and *Tph2* KO) mice, suggesting that EC cells may also participate in CRC progression. This study focused on the 5-HT produced by ENS and its role in CRC tumorigenesis, but it cannot rule out the possibility of the regulation of 5-HT production in the gut by the central nervous system (CNS); maybe it is the crosstalk regulation between the CNS and ENS. In the long run, the relationship between the 5-HT–Wnt axis and CRC progression discovered by Zhu and colleagues may lead to great progress in CRC treatment.

## Author contributions

JJ, MW, SX, ZH, and YJ contributed to writing and editing the manuscript. All authors contributed to the article and approved the submitted version.

## Funding

This work was supported by the National Natural Science Foundation of China (82103210) and the Research Start-up Project by Hangzhou Normal University (4125C50220204106).

## Conflict of interest

The authors declare that the research was conducted in the absence of any commercial or financial relationships that could be construed as a potential conflict of interest.

## Publisher’s note

All claims expressed in this article are solely those of the authors and do not necessarily represent those of their affiliated organizations, or those of the publisher, the editors and the reviewers. Any product that may be evaluated in this article, or claim that may be made by its manufacturer, is not guaranteed or endorsed by the publisher.
